# Proposal for New Method for Calculating Sedimentation Process Efficiency in Water Treatment Plants

**DOI:** 10.3390/ma17133285

**Published:** 2024-07-03

**Authors:** Marian Banaś, Bartłomiej Hilger

**Affiliations:** Department of Power Systems and Environmental Protection Facilities, Faculty of Mechanical Engineering and Robotics, AGH University of Science and Technology, Mickiewicza 30, 30-059 Krakow, Poland; mbanas@agh.edu.pl

**Keywords:** fine-grain materials, sedimentation, water treatment, fractal dimension, sedimentation process efficiency

## Abstract

An important aspect of water treatment is removing fine-grain materials from water. Due to the properties of fine-grain materials, they are difficult to remove from water. During the sedimentation process, which takes place in settling tanks, such materials are removed. The sedimentation process is often accompanied by coagulation and flocculation processes, which form aggregates of particles (flocs) from the fine-grained material particles in a suspension (non-grainy suspension). This kind of suspension (consisting of aggregates of particles or flocs) shows a different behaviour when falling compared with classic grainy suspensions. The main goal and novelty of this article are to propose (and test) a modification of the often used Stokes’ formula with the addition of fractal geometry into the calculation of the terminal velocity of free-falling particles in order to overcome Stokes’ formula’s limitation, thus obtaining the sedimentation process efficiency. Because of this fractal modification, it is possible to use the simple and elegant Stokes’ formula in order to calculate better the terminal velocity of non-grainy particles—aggregates or flocs—and thus obtain the sedimentation efficiency for the whole range of suspensions, both non-grainy and grainy. The results obtained in this article show that the sedimentation process efficiency calculated by using the modified formula based on the fractal geometry morphology of particles (the proposed fractal method) describes and agrees more with the data from the experiment than the sedimentation efficiency calculated only based on particle size (classic method).

## 1. Introduction

In many industrial processes, there is a need to deal with fine-grain materials, because this kind of materials are omnipresent in nature, and in many industrial processes, they are processed in some form or another. One of the ways to process this type of materials is the sedimentation process, which takes place in sedimentation tanks [1]. In order to fully process a fine-grain material, it is necessary to know the granulometric composition of the processed phase [2]. This is vital when designing the settling tanks used in wastewater processing plants, as well as for water renewable processes [3], due to law and state regulation to provide the population with high-quality and safe-for-health potable water. It is important for public health to remove the solid phase from potable water, because the surface of the particles can be inhabited by microorganisms and bacteria, which can be harmful to human health. Infectious diseases caused by pathogens that can be present in water are the most common and widespread health risks associated with potable water [4].

In the water treatment process, removing the solid phase by using coagulation and flocculation processes plays an important role. Due to this process, aggregates of particles and flocs (non-grainy particles) are created from the fine-grained material particles (grainy particles). The aggregates of particles are significantly different from the fine-grained material and form different kinds of suspensions. Grainy particles create grainy suspensions, which consist of compact particles, where the shape (and size) of such particles can be easily described and approximated by Euclidean geometry (e.g., the diameter of a sphere). For non-grainy suspensions, the main solid-phase components are non-grainy particles. This kind of solid-phase particles often have complex, non-trivial shapes and structures, which can be difficult to describe with one parameter (such as a diameter). For such aggregates of particles, fractal geometry can be used to describe the particle’s complex shapes in a simple way, using a small number of parameters [5].

The granulometric composition of fine-grain materials can be understood as both the shape of the particles and their size (or even a size distribution). In many descriptions of the processing of fine-grain materials, the assumption of a spherical shape is used due to the simplicity and elegance of describing the falling particles (e.g., in the form of Stokes’ formula). However, particles of the solid phase present in nature are very rarely spherical, and most often, they have a shape significantly different from a sphere [6]. Sometimes, they can have regular shapes, such as polyhedrons, but usually, they are irregular geometric solids, which can even be aggregates composed of smaller particles (for example, flocs which form in water during water treatment). In many cases, particle size is a key parameter controlling their behaviour—i.e., the settling velocity of the particle [7,8,9]—in various processes (sedimentation process and flotation process) [10,11]. Usually, there is never a set of identical (in diameter) particles in a suspension (monodisperse suspension). In the case of real suspensions observed in most processes, the solid phase is polydisperse—i.e., the particles have different sizes. Particles sizes in such sets are described by using the parameters of statistical functions of distributions of random variables [12] (e.g., log-normal distribution, Gamma distribution or Rosin–Rammler distribution).

### 1.1. Sizes of Particles of Irregular Shapes

The size of a single particle is clearly described in the case of a spherical shape (this is the diameter of such a particle) or for regular solids (e.g., cubes, cuboids, etc.)—the length of the edge. However, for particles characterised by a regular shape, e.g., a cuboid, three numbers (length, width and height) would be needed to fully describe the size of a single particle. Moreover, in the case of particles with compact but irregular shapes, it is impossible to describe their size in the form of a few numbers. Because of that, the particle size needs to be determined, preferably as a single variable. For this reason, the concept of equivalent size or equivalent diameter of a sphere is often introduced [13]. This concept is closely related to the chosen particle size determination method or particle analysis process. The equivalent diameter is the diameter of an imaginary sphere with a selected parameter (projected area, total area, volume, etc.) being the same as in the case of the real considered non-spherical particle. The most commonly used equivalent diameters for particles of fine-grain materials [14] are shown in Table 1.

Characteristic sizes for particles of irregular shape can be used only after the appropriate orientation of the plane of the observed particle (e.g., for a plane perpendicular to the direction of observation or direction of flow of medium). Geometrical features such as the cross-sectional area or circumferential length of the particle can also be used as an equivalent size for the particle. In the case of free-falling particles or particles flowing through the measuring cell of the analyser, irregularly shaped particles tend to arrange themselves in such a way that the smallest surface area is in the direction of the flow. That is due to the fact that in this position, the particles will have the least flow resistance.

In industrial processes, we rarely deal with particles with a spherical shape or with the shape of regular solids. Information about the particles’ shape is necessary to characterise the distribution of such particles (description of granulometric characteristics). Shape factors are proportions of selected geometric features (e.g., length of the longest chord, cross-sectional area, surface area or volume) or physical properties determined by the shape of the particles. Determining the numerical value of these coefficients is relatively complicated. It requires the tedious processing of measurement results for a large number of individual particles, and because of that, the information about the collection of particles is inaccurate or impossible to determine. The more important correction factors are presented in Table 2.

In the case of particles with a complex structure (in the form of a cluster of aggregates of smaller particles or flocs), their description, especially in the quantitative form of their structure, is a non-trivial task. For this purpose, fractal geometry can be used. It has already been used successfully to describe numerous phenomena and objects (also non-physical ones) with stochastic properties but not completely irregular. Fractal geometry uses the concept of a fractal, where selected properties are repeated and transferred in many scales [15]. This scaling ability is described by a fractal dimension which has a sense similar to the topological dimension and can be tuned for a given fractal object by the so-called fractal prefactor. The scaling of properties is described by the fractal power law, expressed by Equation (1):

(1) $$A(x)=k\cdot x^{D_{f}}$$

where A(x) describes the selected physical feature of a fractal object, x is the selected quantity describing a fractal object, k is the fractal prefactor, and D_f_ is the fractal dimension.

**Figure 1:**
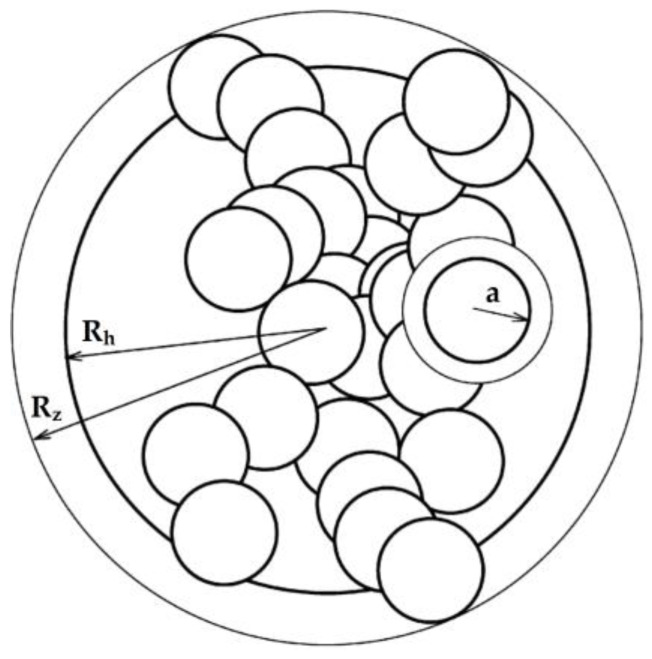
Structure of a non-grainy particle (aggregate).

The particle with a complex structure can be treated as a fractal object—an aggregate consisting of elementary particles. The spatial arrangement of the elementary particles can be described by a fractal dimension recurring on several scales. A diagram of such aggregate is shown in Figure 1.

To describe a fractal particle, one should specify the size of the elementary particle, the fractal dimension (describing the distribution at many scales) and the selected substitute size of the aggregate [16]. In this model, several basic dimensions describing the geometric size of a particle can be distinguished. For the sake of simplicity, it is assumed that the individual elements of the aggregate structure are reduced to the radii of certain equivalent spheres. The most important of these sizes are the following:

- The radius of the basic particle (primary particle)—a or $r_{p}$—for the presented model in Figure 1. For simplification, it is assumed that all of the basic particles in the aggregate have the same radius and have the shape of a sphere. An additional assumption is that the density of the basic particles is isotropic and constant for all of them.
- Outer radius—$R_{z}$—the radius of the smallest ball, in which the floc under investigation can be inserted—the radius of the bounding (circumscribed) sphere on the agglomerate. It can be determined by several direct (optic) methods, such as optical microscopy. An alternate name for the outer radius is the collision radius.
- Stokes–Einstein radius (hydrodynamic radius)—$R_{h}$—the radius of a sphere that has the same hydrodynamic drag as the floc under investigation. One important assumption is that the equivalent sphere is impermeable and only the drag of the flow is taken under consideration. The influence of advection flow through the volume of the particle is ignored. The Stokes radius allows us to determine the behaviour of the agglomerate as it is freefalling in the fluid.
- Radius of gyration—$R_{g}$; this radius describes the distribution of mass of basic particles around the centre of gravity of the aggregate. It is defined as the mean square of the distance of the basic particles from the centre of mass of the aggregate, weighted against the mass of these particles.

The fractal particle can exhibit many fractal-scaled properties. By using the power law, it is possible to describe its density, the free-falling drag force of the particle or its settling speed in a viscous environment, i.e., during the sedimentation process. One of the properties, fractal-scaled, is the number of basic particles in the aggregate, described by Equation (2):

(2) $$i=k{\cdot \left(\frac{d}{d_{p}}\right)}^{D_{f}}$$

where i is the number of basic particles, d is the diameter of the aggregate and d_p_ is the diameter of the basic particle. The mass of the aggregate can then be calculated by Equation (3):

(3) $$M=k{\cdot \left(\frac{d}{d_{p}}\right)}^{D_{f}}$$

M is the mass of the whole aggregate, and k is the fractal prefactor. The conclusion that can be deducted from dependence (3) is a relationship between the density of the aggregate ρ and its fractal dimension, and this is presented in Equation (4):

(4) $$\rho =k{\cdot \left(\frac{d}{d_{p}}\right)}^{D_{f}-3}$$

**Figure 2:**
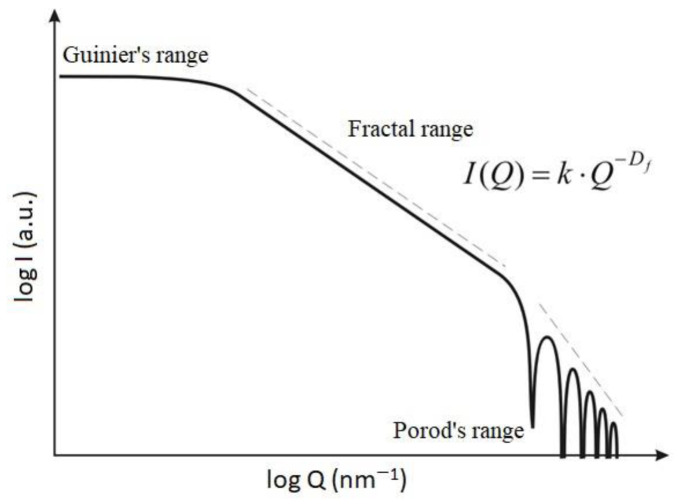
Scattered light intensity in the function of the wave number.

Obtaining granulometric characteristic and in particular the fractal dimension of fractal aggregates can be carried out in several ways. These include methods based on the analysis of radiation scattering or the analysis of obscuration values in laser diffraction, image or particle settling velocity analysis. One of such methods is the low-angle laser light-scattering (LALLS) method. In this method, the results of measurements obtained from a laser diffractometer can be used to determine the fractal dimension. For this purpose, the fact that the intensity of the radiation scattered on particles of the solid phase depends on the wave number and on functions that describe the influence of the shape of the basic particle (shape factor) and the spatial distribution of basic particles inside the floc (structure factor) is used. This relationship is represented by Formula (5):

(5) $$I\left(Q\right)=I\left(0\right)\cdot P\left(Q\right)\cdot S(Q)$$

where I(Q) is the intensity of radiation at the angle of 0°, P(Q) is a shape factor and S(Q) is a structure factor. Relationship (5) is presented in Figure 2 on a double logarithmic scale. Three ranges can be distinguished in it (Guinier, fractal and Porod), with characteristic elements scattering radiation for each of the ranges [13].

In the Guinier range, radiation is scattered on large structures, such as aggregates and flocs (the shape function plays the smallest role in radiation intensity; the structure function has the greatest impact). In the fractal range, both functions affect the intensity of scattering that occurs on fractal aggregates. In the Porod range, the scattering occurs mainly on the basic particles of the suspension (the structure function, in this case, does not significantly affect radiation intensity, and the shape function has the greatest impact).

In the fractal range, the power law, which scales the dependence of the scattered light intensity with the wave number of the scattering vector, can be applied. This law is presented in Equation (6):

(6) $$I\left(Q\right)=k\cdot Q^{{-D}_{f}}$$

Formula (6) is the basis for determining the fractal dimension of particles from data from diffraction measurements. After taking the logarithm of Equation (3), a quasi-linear approximation can be calculated, represented by Equation (7):

(7) $$\log I={-D}_{f}\cdot \log Q+\log k$$

Because of this, it is easy to determine the fractal dimension numerically, obtaining it from a graph created on the basis of Equation (5) from the slope coefficient of a straight line located in the fractal range of this graph.

### 1.2. Sedimentation Process

The basic point for sedimentation process modelling is to consider the settling velocity of a particle in a suspension and consider the particle’s size. In theoretical calculations, a number of assumptions are made to approximate the properties of the falling particle and the properties of the medium in which it falls. Usually, it is assumed that the particle has the shape of a sphere; the medium in which the free fall of the particle takes place is stationary, homogeneous and isotropic; and the viscosity and density of the medium are constant in time and space. The motion of a falling particle is considered in one-dimensional space.

The equation of the free fall of a particle in a fluid with such assumptions has the following general form of Equation (8):

(8) $$m\frac{d_{\vec{v_{s}}}}{d_{t}}=\sum_{i=1}^{n}{\vec{P}}_{i}$$

**Figure 3:**
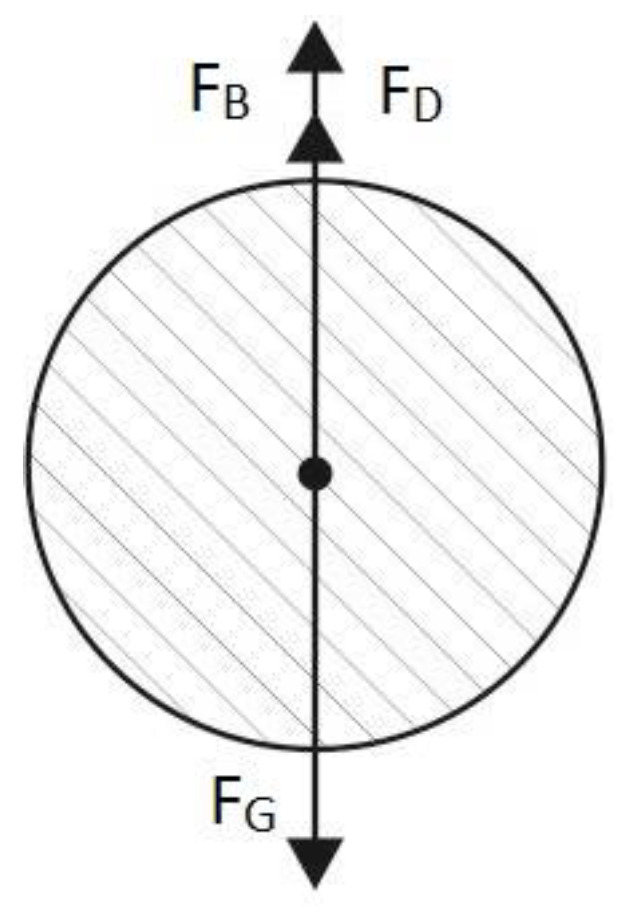
Forces which are under consideration during the free fall of a particle.

The left side of the equation represents the inertial force acting on the particle. The right side is the sum of all active forces acting on the particle in the solid phase. This situation is shown in Figure 3. Moreover, the forces are as follows: the buoyant force of the particle F_B_; the force of resistance to the movement of the particle F_D_—drag force; and the force of gravity F_G_ affecting the particle.

The force of gravity is a force which results from the action of gravity on the particle. It is equal to the mass of the particle and the intensity of the gravitational force field (gravitational acceleration g); it is shown in Equation (9):

(9) $$F_{G}=m\cdot g$$

The force of buoyancy is the force resulting from the pressure difference on the lower and upper surfaces of the particle. The buoyant force equals the weight of the fluid displaced by the particle. This force can be expressed as the product of the volume of the particle V and the specific gravity of the fluid γ. For a spherical particle, the volume can be expressed by the formula for the volume of a sphere with diameter d, and the specific gravity can be expressed by the density of the displaced fluid ρ_f_ and the gravitational acceleration g. The force of buoyancy can be calculated as

(10) $$F_{B}=V\gamma =\frac{\pi d^{3}}{6}{\cdot \rho }_{f}\cdot g$$

The drag force equals the drag area A and the dynamic pressure p_dyn_. The drag force also depends on many factors. The influence of these factors can be considered with the drag coefficient ψ. For a ball, the surface of resistance A will be the projection area of the ball on a plane perpendicular to the direction of motion. Dynamic pressure will represent kinetic energy relative to the mass of a unit volume of fluid. Hence, the drag force of the particle motion can be written in the form of Equation (11):

(11) $$F_{D}=\psi \cdot A\cdot p_{dyn}=\psi \cdot \frac{\pi d^{2}}{4}\cdot \frac{\rho_{f}{v_{s}}^{2}}{2}$$

By assuming that the particle’s falling velocity is constant, the left-hand part of Equation (8) is equal to 0, since the derivative of the velocity with respect to time will be equal to 0:

(12) $$v=const\;\to \frac{d_{\vec{v_{s}}}}{d_{t}}=0$$

The equation of three forces can be written as presented in Equation (12), since the forces are parallel to each other:

(13) $$F_{D}=F_{G}{-F}_{B}$$

After substituting Equations (9)–(11) into Equation (13) and taking into account that the mass of the particle m can be expressed as the product of the volume of the sphere and the density of the particle, the following equation is obtained:

(14) $$\psi \cdot \frac{\pi d^{2}}{4}\cdot \frac{\rho_{f}{v_{s}}^{2}}{2}=\frac{\pi d^{3}}{6}{\cdot \rho }_{s}\cdot g-\frac{\pi d^{3}}{6}{\cdot \rho }_{f}\cdot g$$

After the transformations, the following dependence on the particle settling velocity is obtained:

(15) $$v_{s}=\sqrt{\frac{4}{3}\frac{\left(\rho_{s}-\rho_{f}\right)}{\rho_{f}}\frac{d}{\psi }}$$

From Equation (15), an important property emerges: the particle’s settling velocity in the fluid depends on the size of the particle and its density. In Formula (15), there is a drag coefficient ψ, which depends on the properties of the fluid (density and viscosity), as well as on the size and speed of grain settling. The method of drag coefficient calculation was given by Stokes. To determine this coefficient, it is required to know the Reynolds number, which represents the ratio of inertial forces to viscous forces. The Reynolds number is given by Equation (16):

(16) $$Re=\frac{v_{s}d}{v}=\frac{v_{s}d\rho_{f}}{\mu_{f}}$$

where v is the kinematic viscosity of the fluid and μ is the dynamic viscosity of the fluid. The general equation of motion of a viscous fluid is as follows:

(17) $$\rho_{f}\frac{\partial u_{f}}{\partial t}+\rho_{f}u_{f}\left(\nabla u_{f}\right)=-grad\;p_{f}+\mu \nabla^{2}u_{f}+\rho_{f}g$$

where p_f_ is the pressure of the fluid, ∇ is the Nabla operator and ∇^2^ is the Laplace operator.

By assuming that the flow velocity is constant and neglecting the effects of inertia forces, the equation is simplified to the form

(18) $$grad\;p_{f}=\mu \nabla^{2}u_{f}+\rho_{f}g$$

This form of the equation of fluid flowing around a sphere with a flow velocity of u_f_ was considered by Stokes. The velocity of the fluid flowing around a spherical particle u_f_ can be interpreted as equal to the velocity of the spherical particle in stationary fluid (or the terminal velocity of the free-falling spherical particle v_s_).

The equation for calculating the drag force F_D_ is one of the results of Stokes considerations. The drag force can be obtained from Equation (19):

(19) $$F_{D}=3\pi \mu dv_{s}$$

This relationship is called Stokes’ law: In a steady flow, the resistance force of a stationary fluid against a falling particle is proportional to the terminal velocity of the particle vs and to its size d.

On the other hand, the drag force of a viscous medium, i.e., the force opposing the flow of a particle, according to the Newton equation, is given by Formula (20):

(20) $$F_{D}=\Psi \cdot A\frac{v_{s}^{2}}{2}\rho_{f}$$

A is the projected area of the perpendicular surface of the particle to the movement of the particle. By comparing Equations (19) and (20), one can obtain Equation (21):

(21) $$3\pi \mu dv_{s}=\Psi \cdot \frac{{\pi d}^{2}}{4}\frac{v_{s}^{2}}{2}\rho_{f}$$

From this comparison, it is possible to calculate the drag coefficient Ψ, correct for the so-called “small” Reynolds numbers $10^{-4}<Re<0.25$:

(22) $$\Psi =\frac{24}{Re}$$

In matters related to the design of environmental protection devices and processes, the right boundary of Formula (22) is extended to Reynolds number Re=2, assuming that in practical matters, the flow around the particle will slightly differ from the laminar flow and the resulting errors are not significant. This formula is called Stokes’ formula, and it connects the particle velocity with the basic material parameters of said particle and the properties of the medium in which the particle is freefalling. Stokes’ formula is presented in Equation (23):

(23) $$v=\frac{1}{18}\frac{\left(\rho_{s}-\rho_{l}\right)}{\mu }gd^{2}$$

where v is the settling velocity of the particles in the suspension; ρ_s_ and ρ_l_ are the particle and fluid density, respectively; g is the gravitational acceleration; μ is the dynamic viscosity coefficient; and d is the diameter of the free-falling particle. Stokes’ formula and its modifications are very often used in the modelling of phase separation process efficiency. In the case of suspensions consisting mainly of spherical particles, the size of the falling particles can be described by the selected equivalent diameter [17].

However, when the particles are in the form of agglomerates or flocs, another way of determining the size of such particles is needed. Fractal geometry is used in such cases, which, using the theory of self-similarity, can describe the shape and morphology of particles that are self-similar on different scales [18].

By using fractal geometry, it is possible to describe the settling speed of particles with irregular shapes, significantly different from a sphere, clusters of finer particles and even flocs. The most important modifications to Stokes’ formula using fractal dimensions [13] are presented in Table 3.

### 1.3. Phase Separation Efficiency—The Classical Model

Several models describing the sedimentation process are available in the literature. One of them, which is very often used for determining the theoretical efficiency of phase separation, is the classic model of suspension sedimentation proposed by Kowalski [25]. In this model, the size of the boundary particle is related to the particle size distribution of the suspension with a given density function f(d). The boundary particle is the smallest particle that will be separated from the suspension under steady-state flow conditions or the smallest particles moving at the lowest velocity that will guarantee the separation of these particles from the suspension under steady-state conditions (terminal velocity). Taking into account Camp’s theory, sedimentation efficiency can be presented in the form of Equation (24):

(24) $$\eta \left(d_{g}\right)=1-\int_{0}^{d_{g}}f\left(d\right)dd+\frac{1}{d_{g}^{2}}\int_{0}^{d_{g}}d^{2}f\left(d\right)dd$$

where f(d) is the function of the density of the selected statistical particle size distribution in the suspension, d_g_ is the size of the boundary particle, d is the particle size (particle diameter for spherical particles). To describe the size of the particles in the suspension, a log-normal distribution with the density function can be used, as shown by Equation (25):

(25) $$f\left(d\right)=\frac{1}{\sqrt{2\pi \sigma d}}exp\left\{-\frac{1}{2}{\left(\frac{\ln d-m}{\sigma }\right)}^{2}\right\}$$

where m is the logarithm of the average particle size and σ is the standard deviation of the mean. After the analytical solution of the integrals in Equation (24), the formula expressing sedimentation efficiency from the material values (boundary grain size and granulometric distribution parameters expressed in the form of a log-normal distribution) is obtained, presented by relationship (26):

(26) $$\eta \left(d_{g}\right)=1-\phi \left(\frac{\ln d_{g}-m}{\sigma }\right)+exp\left\{2\left[\sigma^{2}-\left(\ln d_{g}-m\right)\right]\right\}\cdot \phi \left(\frac{\ln d_{g}-m}{\sigma }-2\sigma \right)$$

where ϕ(x) is the value of the distribution function of the normal distribution. The size d_g_ appearing in Formulas (24) and (26) is the characteristic size of the particle and can be associated with the terminal velocity through Hazen’s theory. According to this theory, the boundary particle settling velocity is numerically equal to the value of the surface load of the settling tank, i.e., the quotient of the suspension stream fed to the settling tank and the sedimentation surface (the projection surface of all elements involved in the sedimentation process in the settling tank onto the horizontal plane of the settling tank). This theory is presented by dependence (27):

(27) $$q=\frac{Q}{S}=v(d_{g})$$

where q is the surface load (or hydraulic load), Q is the stream of the suspension fed to the settling tank, S is the total area of all elements in the settling tank projected onto a horizontal plane (parallel to the liquid surface) and v(d_g_) is the boundary particle terminal velocity. The surface load index can describe the sedimentation process under both static and flow conditions. Under static conditions, the indicator is most often expressed in the form [m/h] and carries information about the settling speed of boundary particles at a given moment of the process. On the other hand, under flow conditions, the size of the surface load q is usually expressed in the unabbreviated form [m^3^/m^2^h]. It contains information about the volume of the suspension that is processed per 1 m^2^ of the settling tank surface.

In the classic model, the particle terminal velocity (as a function of the surface load) is calculated from the classic Stokes’ Equation (23) in which only the particle diameter is taken into consideration.

### 1.4. The Fractal Model

The classic model works very well for suspensions in which particles of the solid phase resemble spheres (grainy suspensions) and is consistent with the empirical results carried out in model settling tanks for various suspensions [26]. Unfortunately, in the case of suspension with particles of irregular and developed structure, for example, flocs obtained during the coagulation process (non-grainy suspensions), that model does not coincide with empirical results. In order to better match the empirical results with the results of laboratory tests, another way of describing the size distribution of particles falling in a fluid could be used in such cases. One way is to use the mentioned fractal geometry to characterise the shape of falling particles in the classic model. The development of the classic model with fractal geometry made it possible to apply it to suspensions in which the particles are non-grainy in nature (coagulated particles, aggregates of particles and flocs). Such particles can be described by fractal geometry in the form of fractal aggregates presented before in Section 1.1.

In the proposed fractal model of the sedimentation process, particles of the solid phase can be treated as aggregates composed of small elementary particles of a granular nature (basic particles), which create an extensive spatial structure described by the fractal dimension. The schematic model of such fractal particles is shown in Figure 1.

There are many formulas that take into account the influence of the particle structure described by the fractal dimension on the terminal velocity, and some of them were presented before in Table 3. One of such modifications could be the formula represented by Equation (28):

(28) $$v=k\frac{g}{18\mu }{(\rho }_{sp}-\rho_{f})d_{p}^{{3-D}_{f}}d^{D_{f}-1}$$

where k is a coefficient that includes the fractal prefactor as well as the influence of shape and resistance on particle settling, d_p_ is the size of the basic particles which the particle aggregates in the suspension are made of, d is the size of the particles of the solid phase in suspension and D_f_ is the fractal size of the particles of the solid phase of the suspension. The value of the fractal dimension of suspension particles can be determined in several ways. One of the fastest methods is the laser diffraction method for static low-angle laser scattering (LALLS), as mentioned in Section 1.1. The data obtained during laser diffraction tests also allow us to determine the size of the basic particles. For the model, the values of the parameters of the particle size distribution of the solid phase are best determined by using methods in which the physical principle of measurement uses the phenomenon of particle settling. One of such methods is the sedimentation balance method, thanks to which it is possible to obtain the parameters m and σ in the case of a normal distribution for the adopted method of describing the boundary particle settling velocity (e.g., Equation (23) in the case of grainy suspensions or relation (28) in the case of non-grainy suspensions).

## 2. Materials and Methods

### 2.1. Materials

Laboratory studies were conducted on bentonite samples to determine the differences between methods of calculating the sedimentation efficiency (using the classic and fractal models). Bentonite was selected because it can be one of the pollutants originating as runoff from land and present in raw water from rivers or lakes used in water treatment plants. Also, the shape of bentonite particles is complex and unusual: they look like elongated discs or plates and often form agglomerates [27], which was also an important factor for selecting bentonite. These agglomerates behave similarly to agglomerates created during the process of coagulation due to the natural auto-coagulation process of basic grainy particles, even without using additional coagulants.

Three samples of bentonites from Slovakia in this study are referred to as S1, S2 and S3. Samples were characterised by various procedures and tests, which are described below. Before tests, the bentonite samples were dried for 24 h at 105 °C. Big bulks were crushed in a mortar to obtain fine solid phase, which was then used to prepare suspensions.

### 2.2. Methods and Procedures

Particle size distribution was determined with the use of a laser diffraction analyser—Malvern MasterSizer 2000E, Malvern Instruments, Worcestershire, UK. Bentonite samples were added to demineralised water, and after several minutes of ultrasonic treatment and fast stirring, particle size distribution was obtained for each sample. The measurement parameters, such as Particle Refractive Index (PRI) and Adsorption (Ad), for all three bentonites were the same: PRI = 1.61 and Ad = 0.01. Additionally, it is possible to obtain fractal dimensions of suspensions on that apparatus. It was accomplished by using the LALLS method. The raw values read off the analyser were recalculated according to the yield of the light intensity and scattering angles based on tabulated parameters for particular sensors provided by Malvern company. Such analysis was conducted in order to find out the fractal dimension of the particles and test the fractal model of the sedimentation process. The method used is described in detail in Section 1.1.

The SEM image of the main mineral components of the micro area domain was obtained by using a high-resolution scanning electron–ion microscope, FEI Quanta 250 FEG SEM, FEI, Hillsboro, OR, USA. The image was obtained at 4000× magnification under high vacuum.

The density measurement of the solid phase of the bentonite suspension was determined by using the MicroMeritics Accu Pyc 1330 v1.00 helium pycnometer, MicroMeritics, Norcross, GA, USA. This device allows us to determine the volume of the analysed sample by measuring the pressure and volume of helium (at a specific temperature) and then, based on the mass of the sample, to determine its density.

Turbidity was measured with a Turbidirect, Tintometer, Amesbury, UK, apparatus (Lovibond) according to norm EN ISO 7027 [28] by nephelometric means; the infrared light source (IR LED operating at λ = 860 nm) was in a scattering range of 90°. Turbidity was measured between 0.01 and 1100 NTU with an accuracy of ±2% below 500 NTU and ±3% thereafter. All measurements were conducted at a temperature of about 22 °C. The turbidity was used as a convenient parameter for determining the concentration of suspension used in the calculation of sedimentation efficiency during the sedimentation test under flow conditions. The possibility of using turbidity as a substitute for concentration in the sedimentation process was described in detail [29].

The particle size distribution of aggregates and the parameters of log-normal distribution were obtained from the results of sedimentation balance—Mettler Toledo AT460 DeltaRange, Mettler Toledo, Greifensee, Switzerland. The method used in order to obtain these parameters was described in detail [30].

**Figure 4:**
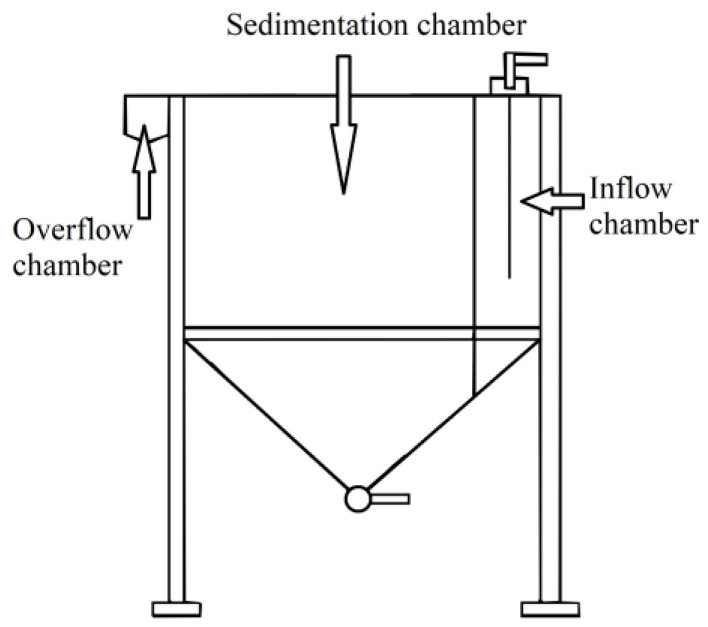
Schematic view of the chambers of laboratory settling tank.

The sedimentation test under flow conditions was conducted in a laboratory settling tank as presented in Figure 4. A laboratory tank is divided into three chambers:

- Priming chamber—the chamber where tested suspension is supplied. In the priming chamber, uniform distribution of the suspension is obtained through a feed tube located over the entire height of the priming chamber and perforated baffle.
- Sedimentation chamber—the space where the multiflux lamella packet is placed. The precipitate is discharged outside the tank through a collector in the shape of funnel, which is located in the lower part of the sedimentation chamber.
- Overflow chamber—the chamber in which clear water after the sedimentation process is obtained through V-notch overflow into the overflow connector.

It is possible to insert a multiflux lamella packet inside the sedimentation chamber in cross-current or counter-current variants. For this study, the counter-current sedimentation multiflux lamella packet was inserted. The multiflux lamella packet is made from five boards—four sedimentation plates and one working as a drive plate. The dimensions of the plates are 210 mm × 50 mm, and the inclination angle is set to 60°, the sedimentation area of the multiflux lamella packet is 210cm^2^.

**Figure 5:**
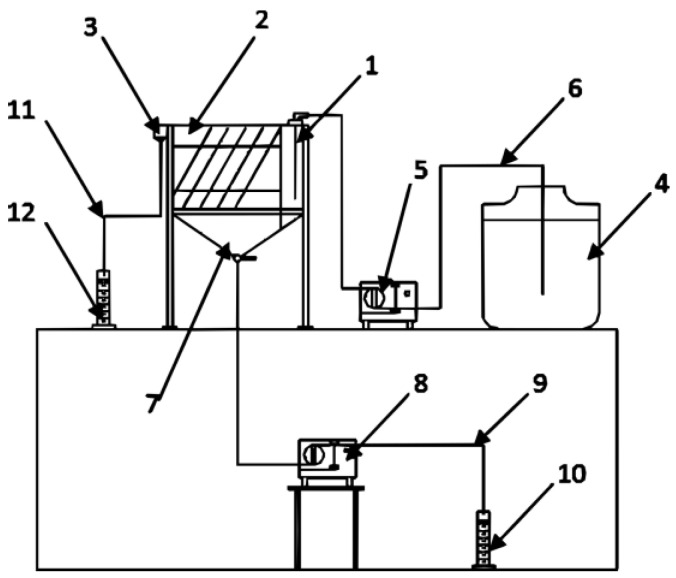
Schematic view of test stand for sedimentation test under flow conditions.

The test stand for the sedimentation efficiency study in the laboratory settling tank is presented in Figure 5. The sedimentation tank is divided into three chambers: the priming chamber (1), the sedimentation chamber (2) and the overflow chamber (3). The prepared suspension is stored in the supply tank (4). The supply tank is equipped with a stirrer for uniformly mixing the prepared suspension. The suspension prepared in this way is transported by a hose (6) through a peristaltic pump (5) to the priming chamber (1), wherein with a feed tube, the suspension is uniformly distributed into the sedimentation chamber (2). The sedimentation chamber is a space where the multiflux lamella packet is inserted and the sedimentation of the solid phase mainly occurs. In the lower part of the sedimentation chamber, the collector is located (7), where collection of the precipitate from the suspension takes place. The concentrated precipitate is transported by a hose (9) and a peristaltic pump (8); then, it is placed in graduated cylinders (10), where the concentration or turbidity of the suspension can be measured. After the sedimentation chamber, clear water flows into the overflow chamber through the V-notch (3) and is also collected by a hose (11) in graduated cylinder (12) for the turbidity test.

When measuring at a low flow rate, a certain part of the solid phase contained in the suspension could remain in the hose, which is used to distribute the suspension into the priming chamber. This is undesirable, because it can interfere with suspension flow and could lead to errors. In order to avoid that situation, the hoses were arranged at a small angle.

The test suspensions with a particular initial concentration (ratio of 5 g/dm^3^) were prepared in a tank equipped with a variable-speed stirrer to uniformly mix the suspension and prevent the solid phase from natural sedimentation. Tests were performed for three surface loads (0.3, 0.5 and 0.8 m^3^/m^2^h) in order to observe the process of sedimentation and determine the sedimentation efficiency for each surface load. In total, three suspensions for three specific surface loads were tested.

After each test, the sedimentation tank, multiflux lamella packet stirrer, hoses and supply tank were washed out and cleaned with water in order to remove the solid phase from all of the equipment. All measurements during the sedimentation test under flow conditions were conducted at a temperature of about 22 °C.

## 3. Results

**Figure 6:**
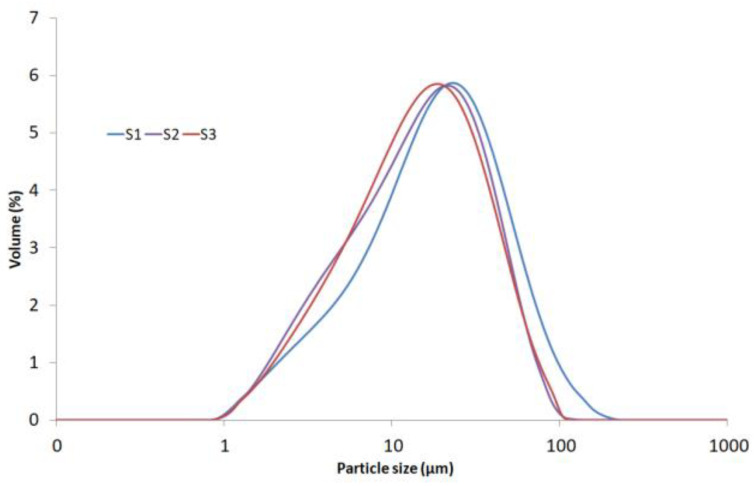
Particle size distributions of the bentonites.

The raw samples of bentonites, consisting of complex aggregates made of basic particles, were tested by using sedimentation balance and a laser diffraction analyser [31,32]. The results are presented in the form of particle size distribution curves (Figure 6), and the statistical parameters of the log-normal distribution are shown in Table 4.

As can be seen in Figure 6, the grain size distribution curves for the bentonites are monomodal in character, with maxima in the range of about 20 μm for S1. The grain size distribution for the bentonites is narrow (0.7 ÷ 100 μm), and the grain compositions of S2 and S3 are more homogeneous than that ofS1. The median values from different analysers are quite comparable with each other. This is also confirmed by the small difference between the data from sedimentation balance, and laser diffraction analysis (Table 4). The fractal dimension for all the samples is very similar, and its values are between 1.83 and 1.86.

**Figure 7:**
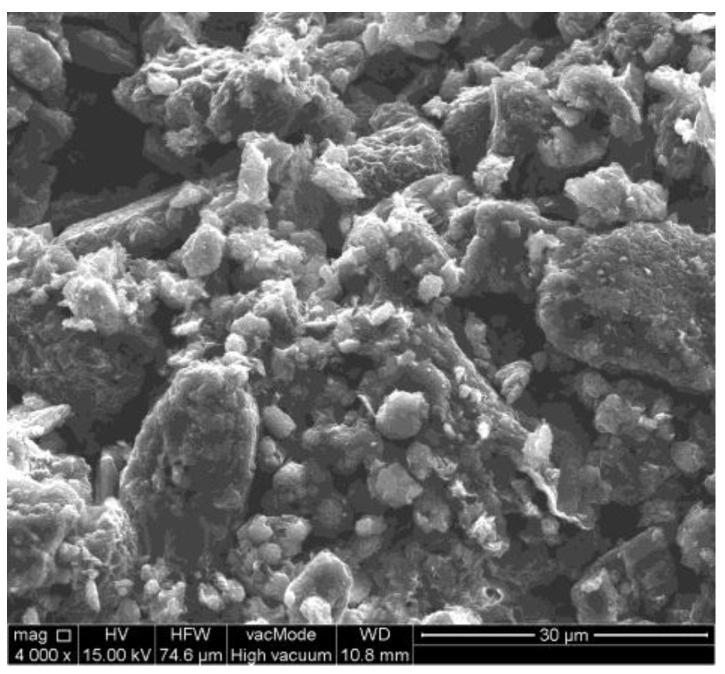
A SEM image of the structure of the bentonite (S2).

The particle size distribution of the basic (primary) particles was obtained after 10 min of ultrasonication (20 kHz). The exposure of the raw suspensions to ultrasonic energy allowed for the breaking up of the structure of aggregates into basic particles. The medians of the basic particles in the samples were as follows: S1—3.5 μm; S2—3.3 μm; S3—3.4 μm. After ultrasonication, the fractal dimension also decreased to about 1.63 for all of the samples. Additionally, as the fractal dimension of these particles is between 1 and 2, it suggests that the basic particles are more in the shape of something two-dimensional—like discs or plates—which is also confirmed by the SEM image of the bentonite S2 particles (Figure 7).

The densities of the tested bentonites (Table 5) were similar, especially for the S1 and S2 bentonites. The third one was denser than the first two (about 5% denser). The density of the bentonites falls into values reported by other studies [33,34]. The density is one of the factors that is necessary to calculate the terminal velocity of the particles.

Calculations of sedimentation efficiency for classic and fractal models were conducted from the data collected during the investigations. The calculations were compared with the results obtained from the sedimentation tests under flow conditions. The efficiency of sedimentation under flow conditions was obtained by calculating the ratio between the turbidity of the cleaned suspension from the overflow stream to the turbidity of the suspension, which was pumped into the settling tank. The values of the calculated sedimentation efficiency for theoretical models and the laboratory experiment are presented in Table 6.

In the calculation of sedimentation efficiency for the classic model, Equation (26) (Section 1.3) and Equation (23) (Section 1.3) were used. In the case of the fractal model, the terminal velocity of particles was calculated by using Equation (28) (Section 1.4), instead of Equation (23).

**Figure 8:**
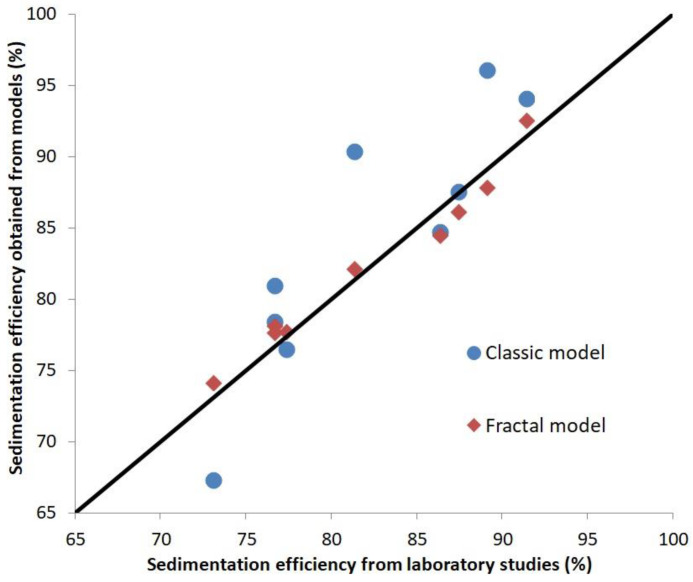
The comparison of the sedimentation efficiency obtained with the models (fractal and classic) and the sedimentation efficiency from the laboratory studies.

The efficiency of sedimentation highly depends on the surface load. For low surface loads (0.3 m^3^/m^2^h), sedimentation efficiency was higher—85 ÷ 90%—than for the higher surface loads (0.5 m^3^/m^2^h and 0.8 m^3^/m^2^h), at about 70 ÷ 80%. The sedimentation efficiency decreases because of the increased suspension flow through the sedimentation tank. The differences between the sedimentation efficiency obtained with the fractal model of sedimentation and the results from the sedimentation tests under flow conditions were smaller (the maximum difference was less than 2 percent points) than those between the classic model and the results from the sedimentation test under flow conditions (the highest difference was about 9 percent points). The comparison of the sedimentation efficiency obtained with the models (fractal and classic) and the sedimentation efficiency from the laboratory studies is presented in Figure 8. The results show that the sedimentation efficiency obtained from the fractal model is more consistent with the sedimentation efficiency from the laboratory studies than that obtained with the classic model.

So, in this new model, fractal dimension is connected with the properties of the particle aggregates such as shape, permeability and thus drag coefficient. Taking these properties into account in the modified Stokes’ formula allowed us to determine a particle terminal velocity more consistent with the laboratory data compared with the classic model, in which a few simplifications were made (mainly that the particle was a sphere). This better approximation works even for uncoagulated suspensions, in which the solid phase is composed of aggregates of fine-grain particles—basic particles. The only disadvantage of such an approach is the fact that the fractal dimension of aggregates and the size of the basic particles need to be found (experimentally).

The proposed model requires several steps in order to calculate the sedimentation efficiency in the settling tank. The steps that should be performed are presented in order as follows:

1. Measurement of several characteristic properties of solid-phase particles in suspension:
  - The particle size distribution of aggregates/flocs in the form of parameters of the log-normal distribution (m and σ);
  - The density of the solid phase of the suspension (and, from the information of the temperature during tests, the values of the density and viscosity of the fluid phase);
  - The size of the basic (primary) particles and the value of the fractal dimension of aggregates/flocs in the suspension.
2. Calculation of the diameter of the terminal particles by using a selected equation between the velocity of the free-falling particles and physico-chemical properties of the particles under consideration (the modified Stokes’ formula—Equation (28)—is used in the proposed method).
3. Calculation of sedimentation efficiency with the use of information on terminal velocity and granulometric properties of the suspension (Equation (26) is used in the proposed method).

The sedimentation efficiency for given surface load obtained in this way can be used in order to model the behaviour of a suspension during the sedimentation process in a sedimentation tank, which is the key point of our new method of calculating sedimentation process efficiency.

## 4. Conclusions

In conclusion, it is possible to use fractal geometry to define the morphology of particles in suspensions, where bentonites or similar minerals (clay minerals) or other non-grainy materials are present. By using the modified Stokes’ formula with fractal dimension, it is possible to describe the behaviour of particle aggregates during the sedimentation process in a sedimentation tank.

The proposed model can better predict the sedimentation process efficiency in a settling tank, in which the sedimentation process of non-grainy suspensions is carried out. The proposed procedure of calculating of sedimentation efficiency can contribute to the development of new solutions in sedimentation devices or the modernisation of existing settling tanks. This can lead to improvements in public health by ensuring high-quality and pollutant-free potable water used in water supply networks.

The proposed model can be used for a variety of non-grainy materials, which can form particle aggregates or flocs in the processes of coagulation and flocculation used in water treatment plants, not only as a result of the coagulation/flocculation process but also in the process of natural auto-coagulation of fine-grained materials such as bentonites.

## Figures and Tables

**Table 1:** Equivalent diameters used in order to describe particles.

Name	Description	Formula
Perimeter diameter	Diameter of a circle with the same perimeter as the considered particle	$d_{c}=\frac{P}{\pi }$
Projection area diameter	Diameter of a disk with the same projected cross-sectional surface as the considered particle	$d=\sqrt[{2}]{\frac{A}{\pi }}$
Surface diameter	Diameter of a sphere with the same surface as the considered particle	$d_{S}=\sqrt{\frac{S}{\pi }}$
Volumetric diameter (equivalent spherical diameter)	Diameter of a sphere with the same volume as considered the particle	$d_{V}=\sqrt[{3}]{\frac{S}{\pi }}$
Surface volume diameter or Sauter mean diameter	Diameter of a sphere with the same ratio of surface area to volume as the considered particle	$d_{SV}=\frac{d_{V}^{3}}{d_{S}^{2}}$
Free-falling diameter	Diameter of a sphere with the same density and free-falling velocity that the considered particle has in the same fluid medium (at the same density and viscosity)	$d_{St}=\frac{18\mu v}{\rho_{s}-\rho_{f}}$
Stokes diameter	As above, but with Reynolds number Re < 0.2	
Feret diameter	Distance between the two parallel planes restricting the object perpendicular to that direction	$d_{F}$
Martin diameter	Length of the chord parallel to a fixed direction, which splits the particle projected area into two equal parts	$d_{M}$
Circumscribing diameter	Diameter of the smallest circle that circumscribes the outline of the particle	$d_{C}$
Inscribing diameter	Diameter of the biggest circle which fits inside the outline of the projected particle.	$d_{I}$

P—perimeter of projection of particle, μm. A—area of projection of particle, μm^2^. S—area of whole particle, μm^2^. ρ_s_—density of particle, kg/m^3^. ρ_f_—density of fluid, kg/m^3^.

**Table 2:** Selected shape factors.

Correction Factor	Description	Formula
Form factor (FF)	Deviation of an object from a circle; it is especially well used for describing “rough” particles. For circular particles, FF assumes the value of one.	$FF=\frac{4\pi A}{P^{2}}$
Three-dimensional aspect ratio (aspect ratio) (AR)	It is very sensitive to the extension of an object, where the more elongated the object is, the larger the value of this coefficient; as above, circles have the AR of one.	$AR=1.0+\frac{4}{\pi }\cdot \left(\frac{L}{B}-1.0\right)$
Elongation factor	The more elongated the shape of a particle, the higher its elongation factor.	$EF=\frac{L}{B}$
Roundness (RD)	It is mainly influenced by the elongation of an object, and it varies between 1 (circle) and 0.	$RD=\frac{4A}{\pi L^{2}}$
Heywood circularity factor	Perimeter divided by the circumference of a circle with the same area. The closer the shape of a particle is to a disk, the closer the Heywood circularity factor is to 1.	$HCF=\frac{P}{2\sqrt{\pi A}}$
Feret factor	Ratio of Feret diameters (horizontal and vertical).	$FE=\frac{d_{FH}}{d_{FV}}$
Malinowska factor	Relationship between perimeter and cross-sectional area of particle.	$RM=\frac{A}{2\sqrt{\pi P}}-1$
Elongated shape factor	Square root of the two second moments of the particle around its principal axis.	$f_{elong}=\sqrt{\frac{i_{2}}{i_{1}}}$
Compactness shape factor	Function of the polar second moment of a particle and a circle of equal area.	$HCF=\frac{A^{2}}{2\pi \sqrt{i_{1}^{2}{+i}_{2}^{2}}}$
Wadell’s spherical factor (sphericity)	Surface area of a sphere of the same volume as the particle divided by the actual surface area of the particle.	$\psi =\frac{S_{Volume}}{S_{grain}}={\left(\frac{d_{V}}{d_{S}}\right)}^{2}$
Brockmann’s dynamic shape factor	Flow resistance of particle and flow resistance of equivalent diameter of a sphere with the same mass as the particle.	$\chi =F_{D}/F_{Dm}$

L—the longest chord of particle projection, μm. B—width of the particle projection, μm. i_1,2_—second moments of particle projection around its principal axis, kg·m^2^. F_D_—drag force on particle, N. F_Dm_—drag force on the particle’s mass equivalent sphere, N.

**Table 3:** Selected formulas for free-falling velocity of fractal particles including fractal dimensions.

Author/s	Comments	Formula
Lin and Lindsay [19]	Number of basic particles, permeability and hydrodynamic radius are used for calculation. ${\left(\frac{R_{h}}{r_{p}}\right)}^{D_{f}-3}$ fragment results from permeability and provides information about the degree of filling of the aggregate with basic particles.	$v=\frac{2g}{9\mu }(\rho_{s}-\rho_{l})r_{p}^{2}{\left(\frac{R_{h}}{r_{p}}\right)}^{D_{f}-3}$
Miyahara [20]	Number of basic particles and density of fractal aggregate are used for calculation. $d_{p}^{3-D_{f}}$ describes the density of the fractal aggregate.	$v=\frac{g}{18\mu }(\rho_{s}-\rho_{l})d_{p}^{3-D_{f}}d^{D_{f}-1}$
Adachi and Tanaka [21]	Similar method to Miyahara’s, where the additional factor α describes the permeability and the shape of the non-grainy particle.	$v=\alpha \frac{g}{18\mu }\left(\rho_{s}-\rho_{l}\right)d_{p}^{3-D_{f}}d^{D_{f}-1}$
Witnterwerp [22]	Flow resistance coefficient from function of Reynolds number and shape factors, which are assumed a priori. α, β—shape factors, where for spheres, α=β=1. Re—Reynolds number.	$v=\frac{\alpha }{\beta }\frac{\left(\rho_{s}-\rho_{l}\right)g}{18\mu }d_{p}^{3-D_{f}}\frac{d^{D_{f}-1}}{1+0.15{Re}^{0.687}}$
Khelifa [23]	Similar method to Winterwerp’s, it works well with polydisperse suspensions. θ—shape factor, where for spheres, it has value1. d_50_—median of polydispersed particles. ϕ—value of dispersion of basic particles.	$v=\frac{1}{18}\theta g\frac{\left(\rho_{s}-\rho_{l}\right)}{\mu }d_{50}^{3-D_{f}}\frac{d^{D_{f}-1}}{1+0.15{Re}^{0.687}}\phi$
Gmachowski [24]	Dependences between value of hydrodynamic radius R_h_ and radius of the aggregate R (radius of circumscribed sphere), where r_p_—radius of basic particle. $n={\left(\frac{R_{h}}{R}\right)}^{D_{f}}{\left(\frac{R}{r_{p}}\right)}^{D_{f}}={\left(\frac{R_{h}}{r_{p}}\right)}^{D_{f}}$	$v=\frac{2}{9\mu }(\rho_{s}-\rho_{l})gr_{p}^{2}n^{\frac{D_{f-1}}{D_{f}}}$
Bushell [13]	This formula includes flow resistance, permeability of particle and the fact that due to permeability of the particle, its flow resistance coefficient is smaller. k—structural coefficient of fractal aggregate. k_corr_—correctional coefficient which includes shape of particle and influence of permeability on flow resistance coefficient.	$v=\frac{k}{k_{corr}}\frac{2r_{p}^{2}\left(\rho_{s}-\rho_{l}\right)g}{9\mu }{\left(\frac{R}{r_{p}}\right)}^{D_{f}-1}$

**Table 4:** Log-normal statistical parameters of the bentonite aggregates.

Kind of Bentonite	Parameters of Log-Normal Distribution			Median (μm)	
	m	σ	R^2^	Sedimentation Balance	Laser Diffraction Analyser
S1	2.84	0.56	0.993	17.1	17.5
S2	2.83	0.47	0.990	16.9	14.2
S3	2.65	0.77	0.990	14.2	13.8

Explanation: m, σ—parameters of log-normal distribution; R^2^—coefficient of determination of parameters of log-normal distribution.

**Table 5:** Density of the bentonites under investigation.

Bentonite	Weight (g)	Average Volume (cm^3^)	Average Density (g/cm^3^)
S1	2.0256	0.7601	2.6650
S2	1.6959	0.6397	2.6511
S3	2.3590	0.8431	2.7980

**Table 6:** Summary of results from sedimentation efficiency models and sedimentation tests under flow conditions.

Sample	Surface Load	Sedimentation Efficiency			Differences in Efficiency of Sedimentation	
		Data from Sedimentation Test	Classical Model of Sedimentation	Fractal Model of Sedimentation		
	q	η_b_	η_mk_	η_mf_	η_b_ − η_mk_	η_b_ − η_mf_
	m^3^/m^2^h	%	%	%	p.p.	p.p.
S1	0.3	91.42	94.12	92.62	−2.70	−1.20
S1	0.5	87.47	87.62	86.20	−0.15	1.47
S1	0.8	76.67	78.46	78.19	−1.79	−1.52
S2	0.3	89.12	96.17	87.88	−7.05	1.24
S2	0.5	81.37	90.46	82.18	−9.09	−0.81
S2	0.8	76.67	81.00	77.70	−4.33	−1.03
S3	0.3	86.38	84.78	84.52	1.60	1.86
S3	0.5	77.39	76.56	77.73	0.83	−0.34
S3	0.8	73.07	67.35	74.18	5.72	−1.11

Explanation: p.p.—percentage point.

## Data Availability

The original contributions presented in the study are included in the article, further inquiries can be directed to the corresponding author.
